# Effects of Drought on the Growth of *Lespedeza davurica* through the Alteration of Soil Microbial Communities and Nutrient Availability

**DOI:** 10.3390/jof8040384

**Published:** 2022-04-10

**Authors:** Dongdong Duan, Feifei Jiang, Weihu Lin, Zhen Tian, Nana Wu, Xiaoxuan Feng, Tao Chen, Zhibiao Nan

**Affiliations:** 1 State Key Laboratory of Grassland Agro-Ecosystems, Center for Grassland Microbiome, College of Pastoral Agricultural Science and Technology, Lanzhou University, Lanzhou 730000, China; duandd17@lzu.edu.cn (D.D.); jiangff19@lzu.edu.cn (F.J.); linwh17@lzu.edu.cn (W.L.); tianzh20@lzu.edu.cn (Z.T.); wunn20@lzu.edu.cn (N.W.); fengxx20@lzu.edu.cn (X.F.); zhibiao@lzu.edu.cn (Z.N.); 2 Institute of Rural Development, Gansu Provincial Academy of Social Sciences, Lanzhou 730000, China

**Keywords:** drought, *Lespedeza davurica*, biomass allocation, fungal and bacterial communities, soil nutrients

## Abstract

*Lespedeza davurica* (Laxm.) is highly important for reducing soil erosion and maintaining the distinctive natural scenery of semiarid grasslands in northwest China. In this study, a pot experiment was conducted to investigate the effects of drought (20% water-holding capacity) on biomass and its allocation, root characteristics, plant hormones, and soil microbial communities and nutrients after *L. davurica* was grown in a greenhouse. Drought reduced the total biomass of *L. davurica* but increased the root:shoot biomass ratio. In addition, drought altered the composition and structure of microbial communities by limiting the mobility of nutrients in non-rhizosphere soils. In particular, drought increased the relative abundances of Basidiomycota, Acidobacteria, Actinobacteria, *Coprinellus*, *Humicola* and *Rubrobacter*, which were closely positively related to the soil organic carbon, pH, available phosphorus, ammonia nitrogen (N) and nitrate N under drought conditions. Furthermore, soil fungi could play a more potentially significant role than that of bacteria in the response of *L. davurica* to drought. Consequently, our study uncovered the effects of drought on the growth of *L. davurica* by altering soil microbial communities and/or soil nutrients, thus providing new insights for forage production and natural grassland restoration on the Loess Plateau of China.

## 1. Introduction

A drought event is a recurring phenomenon in many ecosystems under global climate change, and it is predicted to occur more frequently in the upcoming decades [1,2,3,4]. Drought significantly threatens the structure and function of ecosystems, production and many other aspects of agriculture and human society [5,6,7,8]. Semiarid grasslands occupy vast areas in northwest China and are sensitive to drought [9]. However, water availability is considered a key driver for plant composition and productivity in semiarid grasslands [2,10,11,12,13,14]. In addition, drought is a primary limiting factor for agricultural productivity, plant growth and species distribution in the semiarid regions of northwest China [15]. Moreover, root morphological characteristics have become more important to evaluate the environmental impacts on agriculture in semiarid areas and play a crucial role for the uptake of nutrients and affect the ability of plants to compete in the natural community [16].

In addition, drought and nutrient deficiencies are the essential environmental factors that limit plant growth, interactions and aboveground productivity in semiarid grasslands [8,17]. Drought may have a strong impact on ecosystem processes by affecting the chemical properties of soil and availability of water [18,19,20]. On the one hand, drought has substantial considerable effects on plant growth directly through alterations in the availability of water in the soil and its chemical properties [11,21,22,23]. Alternatively, drought affects the activity, abundance, diversity and community structure of soil microbes [24,25,26,27,28,29,30,31]. It also restrains the uptake of nutrients, which, in turn, indirectly influences the performance of plants [29,32,33]; in addition, soil microbes, as major components of soil ecosystems, which have an amazing amount of variety and abundance [34], participating in many key biogeochemical cycling processes terrestrial ecosystems [35,36,37,38]. Many soil microorganisms benefit plants via reducing pathogen incursions and assisting in the acquisition of nutrients from soil [39]. Drought has impacts on the function of soil microbial communities and their ability to interact with plant roots through a reduction in water availability and carbon and nutrient diffusion in soils, leading to changes in nutrient uptake and enzyme activities [40]. For example, drought can lead to the extinction of less resistant microbial populations, a reduction in microbial biomass, and changes in the composition of microbial communities [41,42,43]. During droughts, soil microbes can also synthesize extracellular polymeric compounds to protect their cells and the local environment [44]. As well, a previous study indicated that drought led to a shift in fungal but not bacterial community composition [45]. The differences in the composition of soil microbial communities, in turn, can impact plant performance [46]. A recent study also reported that drought can alter the soil microbial communities, producing strong legacy effects on the competitive interactions of plants [32]. For example, changes in the soil microbial communities can influence the drought tolerance of subsequent generations of plants [47,48]. However, how drought influences semiarid grassland ecosystems remains poorly understood [49,50].

*Lespedeza davurica* (Laxm.) is a C_3_ perennial leguminous subshrub species, which is primarily distributed in temperate regions of the world [51]. It is one of the dominant species in the natural communities of semiarid grasslands in northwest China [52] and is an excellent natural pasture species owing to its high quality and adaptability [51,53]. The severe soil environment in the semiarid areas of northwest China and the decreased abundance of *L. davurica* caused by grazing [15,54] hamper its ability to reduce water and soil loss and maintain forage production in the Loess Plateau region of northwest China [51,55]. Understanding the growth characteristics of the response of *L. davurica* to drought would help to complete the knowledge of its mechanisms of drought tolerance and help to understand its potential role in the production of forage and recovery of natural grassland in semiarid regions of northwest China.

In addition, drought is expected to alter plant physiology and metabolic pathways [56], and plants are likely to alter the level of their hormones to adapt to resource-limited environments. For example, salicylic acid (SA) and indole-3-acetic acid (IAA) are interacted with jasmonic acid (JA), thus regulating the adaptation of plant to its surroundings [57] and the involvement of chitinase in the drought tolerance of tomato (*Solanum lycopersicum*) when subjected to drought [58]. To date, there have been few studies on the response of *L. davurica* to drought stress. One study found that drought decreased the relative growth rate and relative leaf water content of *L. davurica* but increased the content of proline and malondialdehyde, while the activities of catalase, superoxide dismutase and peroxidase increased first and then decreased with the time of drought stress [59]. However, whether the hormones (such as IAA, SA, JA) and the activity of chitinase in the *L. davurica* roots would relate to the growth and development of *L. davurica* under drought conditions was unknown. Besides, drought decreased the total biomass and the total length, surface, and average diameter of the *L. davurica* roots [8,53]. Recently, a study indicated that drought clearly decreased the photosynthesis and concentration of N in *L. davurica* leaves as well [60]. However, studies on *L. davurica* have primarily focused on growth parameters and have rarely examined the indirect effects of drought on the growth of *L. davurica* by alteration of the soil nutrients and soil microbial communities.

The purpose of this study was to illuminate the effects of drought on the growth of *L. davurica* owing to changes in the microbial communities and/or nutrients in soils that were from non-rhizosphere. To achieve this goal, we tested the following hypotheses: (1) drought would increase the allocation of root biomass by *L. davurica*; (2) drought would reduce the mobility of soil nutrients; and (3) drought would have negative effects on the soil microbial communities.

## 2. **Materials and methods**

### 2.1. Soil Sampling, Processing and Seed Collection

The soils used in the study were collected from the Grassland Research Station of Lanzhou University (LZUGRS), Huan County, Gansu Province, northwestern China (37.12° N, 106.82° E). This region is a typical semiarid monsoon climate at an elevation of 1650 m asl. The mean annual temperature is approximately 7.1 °C, and the average annual precipitation is approximately 360 mm. The soil in this area is classified as Cambisol [61], and water is the main limiting factor for plant growth in this area. Approximately 240 kg of fresh topsoil (10 cm depth) were sampled in April 2018. The soil samples were instantly taken to the laboratory where they were sieved to 5 mm to eliminate large roots and plant residues. All the soils were stored at 4 °C before the greenhouse experiment. The maximum water-holding capacity (WHC) of the soils was determined as previously described [62]. We also measured the soil basic concentrations before the seeds were sown in the greenhouse (Table S1).

*Lespedeza davurica* is one of the predominant species in this grassland and covers an average of 7% of the soil [52]. It is a C_3_ perennial herbaceous semi-shrub of the genus *Lespedeza* of the Leguminosae family and is highly palatable [63]. It also has some excellent characteristics, such as tolerance to drought and barren soil. *Lespedeza davurica* seeds were collected from plants that were growing on roadsides (1–2 km from LZUGRS) in early October 2017 when they were ripe and taken to the laboratory where they were air-dried, cleaned, and stored at 4 °C.

### 2.2. Experimental Design

To test whether and how drought modifies the growth of *L. davurica* by altering the soil microbial communities and/or soil nutrients, we performed a pot experiment in which *L. davurica* seeds that had been pre-germinated for 3 days were sown approximately 1 cm deep. Two different soil water treatments were established, including normal soil moisture (60% water-holding capacity (WHC)) and drought treatment (20% WHC), The soil water treatments were used to match the soil water content of natural grasslands in the Loess Plateau of China [64,65].

*Lespedeza davurica* seeds were surface-sterilized with 70% ethanol for 1 min followed by 1% NaClO for 2 min, rinsed three times with ddH_2_O and germinated on sterile glass beads on 10 May 2018. All the pots were maintained at the normal soil moisture before sowing. On 13 May 2018, the 3-day-old, germinated seeds were carefully sown in plastic pots (16 cm in diameter and 18 cm deep) that contained approximately 4 kg of dry soil. Five seeds were sown in each pot. To reduce the edge effect, the germinated seeds were sown 4 cm from the edge of the pot. For the water treatment per soil water, 12 pots (namely 12 replicates) were established. The greenhouse experiment was duplicated to enable us to independently determine the plant biomass of *L. davurica* after 16 days of growth to calculate the relative growth ratio (RGR) of the plants. Thus, this trial included two soil water treatments × 12 replicates × 2 duplicates for a total of 48 pots. The pots were randomized on greenhouse tables at 60% relative humidity, a day/night cycle of 16/8 h, and a day/night temperature regime of 21/16 °C. All the pots were watered every two days to maintain the appropriate soil moisture content at 20% and 60% WHC by weighing at 18:00. Seedlings that died during the first week were immediately replaced by seedlings that had grown for the same number of days. No fertilizers were used throughout the growth period of *L. davurica*. The pots were randomly repositioned weekly to minimize possible variation from the effects of a microclimate in the greenhouse. The plant RGR in g day^−1^ was calculated as shown in Equation (1) [66]:

(1) $$\mathrm{RGR}=\frac{\ln M_{2}-\ln M_{1}}{t_{2}-t_{1}}$$

where two consecutive harvests at times *t*_1_ (plants grown 16 days) and *t*_2_ (plants grown 106 days) in this study yielded the plant biomass *M*_1_ and *M*_2_, respectively.

The first set of pots was destructively sampled after 16 days. The shoots of each pot were clipped at the soil surface, and the roots were carefully rinsed with tap water in a 60-mesh sieve. The shoots along with the roots were then oven-dried at 70 °C for 48 h to a constant weight and weighed.

The second set of pots was collected for destructive sampling at the end of the experiment, which was 106 days after sowing. The plant height was first measured using a flexible ruler. All the plant aboveground parts of each pot were harvested by clipping the shoots at soil surface. Soil samples were obtained destructively in each pot before the roots were harvested and passed through a 2.0-mm sieve to remove large roots and residue. They were then merged uniformly to obtain a pooled soil sample. Soil samples from the pots of each treatment were randomly divided into three portions. The first portion was used to determine the soil water content. The second portion was stored at 4 °C to analyze the soil chemical properties and microbial biomass carbon. The third portion was transported to the laboratory in an icebox and stored at −80 °C until the soil DNA was extracted. This entailed the mixture of four pots of subsamples in each treatment to obtain a pooled soil sample. Thus, three subsamples in each treatment were used to extract DNA. Finally, the *L. davurica* roots were carefully washed away from the soil with tap water, and the length of primary root was measured with a ruler. A subset of fresh root samples from each replicate was rinsed three times with ddH_2_O, quickly frozen in liquid nitrogen and stored at −80 °C before the contents of indole-3-acetic acid (IAA), salicylic acid (SA), jasmonic acid (JA) and chitinase were measured in the roots. The shoots and remaining roots were placed in paper bags, respectively, and oven-dried at 70 °C for 48 h to a constant weight and weighed.

### 2.3. Soil Chemical Properties and Microbial Biomass

The soil organic carbon was determined by the Walkley–Black method, as described by Nelson and Sommer (1982). Total P and total N were determined by adding 1.65 g of catalyst (CuSO_4_ and K_2_SO_4_ at a 1:10 ratio (*w/w*)) and 5 mL of concentrated sulfuric acid to a 0.5 g soil sample. The sample was then digested for 1.5 h at 420 °C, analyzed on a Smartchem 450 Discrete Auto Analyzer (AMS-Alliance, Rome, Italy). Soil ammonia N (NH_4_^+^-N) and nitrate N (NO_3_^−^-N) were analyzed on a Smartchem 450 Discrete Auto Analyzer (AMS-Alliance) by KCl extraction. The soil available phosphorus was determined by NaHCO_3_ extraction—molybdenum antimony colorimetry as previously described [67]. The soil pH was measured as a soil/water mixture at a ratio of 1:2.5 (*w/v*) on a PHS-3C acidometer (Shanghai Yoke Instrument Co., Shanghai, China). The soil microbial biomass carbon was measured by chloroform fumigation-K_2_SO_4_ soil extraction, as previously described [68].

### 2.4. Plant Hormone Measurements

Approximately 0.2 g samples of fresh roots were used to measure the contents of IAA [69], SA [70], JA [71] and the activity of chitinase [72] in the *L. davurica* roots.

### 2.5. DNA Extraction, PCR Amplification and High-Throughput Sequencing

A Magnetic Soil and Stool DNA Kit (Tiangen Biotech, Beijing, China) was used to extract DNA from 0.5 g soil samples, following the manufacturer’s instructions. The primers ITS1F (5′-CTTGGTCATTTAGAGGAAGTAA-3′) and ITS2R (5′-GCTGCGTTCTTCATCGATGC-3′) were used to amplify the ITS1 region of the fungal rRNA. The V3-V4 regions of 16S rRNA were amplified using the 338F (5′-ACTCCTACGGGAGGCAGCAG-3′) and 806R (5′-GGACTACHVGGGTWTCTAAT-3′) primer set. Each sample was amplified in triplicate using an ABI Gene Amp^®^9700 PCR system (Applied Biosystems, Foster City, CA, USA) in a 20 μL reaction system. PCR reactions of the DNA from soil fungi were performed in a final volume of 20 μL, in which 10 ng template DNA, 0.8 μL of forward primer (5 μM), 0.8 μL of reverse primer (5 μM), 2 μL buffer (10×), 2 μL dNTPs (2.5 mM), 0.2 μL rTaq polymerase (TaKaRa, Dalian, China), 0.2 μL bovine serum albumin (BSA), and ddH_2_O had been added to a final volume of 20 μL. The following thermal cycling conditions were used: an initial denaturation at 95 °C for 3 min, followed by 37 cycles of denaturation at 95 °C for 30 s, 55 °C for 30 s, 72 °C for 45 s, and a final extension at 72 °C for 10 min. The PCR reactions of the DNA of soil bacteria were performed in the same final volume of 20 μL with 10 ng template DNA, 0.8 μL of forward primer (5 μM), 0.8 μL of reverse primer (5 μM), 4 μL FastPfu Buffer (5×), 2 μL dNTPs (2.5 mM), 0.4 μL TransStart FastPfu DNA Polymerase (Transgen Biotech Co., Ltd. Beijing, China), 0.2 μL BSA, and ddH_2_O to 20 μL. The same thermal cycling conditions were used as described above for the fungi, except that the 27 cycles of denaturation were used for the 16S rRNA. After amplification, 3 μL PCR of product was detected on a 2% agarose gel. The PCR products were purified using an AxyPrep DNA Gel Extraction Kit (Axygen Biosciences, Union City, CA, USA). The samples were sequenced on a 300PE Illumina MiSeq platform (Illumina, Inc., San Diego, CA, USA) by Majorbio Bio-Pharm Technology Co., Ltd. (Shanghai, China).

### 2.6. Bioinformatics Analysis

Low-quality raw sequences (average phred quality score Q < 20 or sequence lengths < 200 bp) were removed, and the chimeras were filtered. After removing the chimeras, all the effective tags of all the samples were clustered by Uparse (v. 7.0.1090), and high-quality sequences were clustered into operational taxonomic units (OTUs) with a 97% similarity [73]. The representative sequence for each OTU was screened for further annotation. For each ITS representative sequence, the UNITE database [74] was used on the basis of the BLAST algorithm that was calculated by QIIME (v. 1.9.1) to annotate the taxonomic information. In contrast, the Silva Database [75] was used on each 16S rRNA representative sequence based on the RDP classifier (v. 2.11). All the sequence numbers of each sample were normalized to an even number of sequences per sample (52,778 and 28,260 sequences per sample for ITS and 16S rRNA, respectively) for further analysis. α-Diversity, including the Chao 1 and Shannon indices, and β-diversity were computed with Mothur (v. 1.30.2) and QIIME (v. 1.9.1), respectively. A principal co-ordinates analysis (PCoA) was used to visualize the similarity of fungal and bacterial compositions among sampling groups at the OTU level using Bray–Curtis dissimilarities by the *cmdscale* function via the “vegan” package in R. The variance inflation factor (VIF value < 10) was analyzed to screen the environmental factors used for the subsequent redundancy analysis (RDA) owing to the limited number of samples. The RDA was used to reveal the relationships between soil microbial communities and soil properties, which was conducted by the *rda* function via the “vegan” package in R. To further discern the relative importance of drought and the soil property variables for the soil fungal community assembly, a variation partitioning analysis (VPA) was conducted using the “varpart” function of the “vegan” package in R, and the adjusted *R*^2^ values were used to determine the proportion of variation in soil fungal community explained by the fitted model [76]. The bioinformatics analysis described above was performed through an in-house pipeline that was built on the online Majorbio I-Sanger Cloud Platform (http://www.majorbio.com, accessed on 7 September 2021).

### 2.7. Statistical Analysis

All the statistical analyses were performed using R v. 3.5.1 [77]. The normal distribution of data and homogeneity of variance were checked by Shapiro–Wilk and Levene’s tests, respectively. All the data were log-transformed as necessary to meet the standards for normality. Differences between the control and drought were determined by a Tukey’s HSD test (*p* < 0.05).

## 3. Results

### 3.1. Plant Biomass

**Figure 1:**
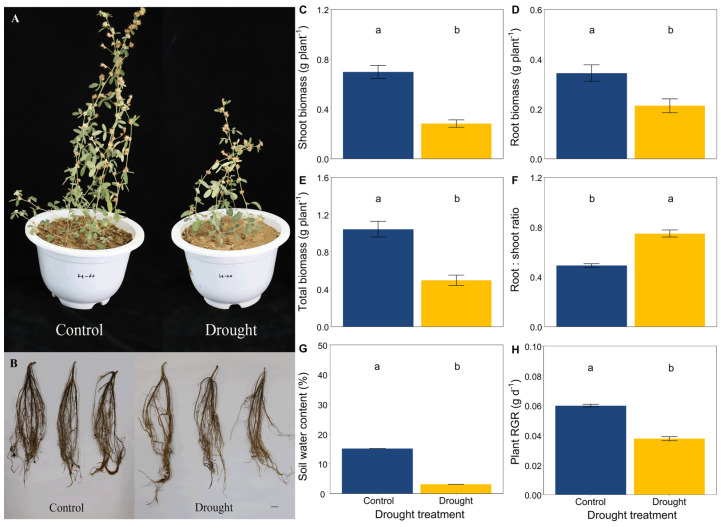
Effects of drought on (**A**) aboveground, (**B**) roots, (**C**) shoot biomass, (**D**) root biomass, (**E**) total biomass, (**F**) the root:shoot biomass ratio of *L. davurica*, (**G**) soil water content, and (**H**) plant relative growth rate (RGR). Black bar = 2 cm in plot B. Mean ± SE are shown (n = 12). Within each panel, different lowercase letters indicate significant differences at *p* < 0.05 (Tukey’s HSD).

Drought significantly limited the growth and development of *L. davurica* by the end of this trial (Figure 1A,B). Drought significantly decreased the height of *L. davurica* but increased the length of its primary root (Figure S1). The soil water content and plant RGR were significantly lower in drought conditions compared with the control (Figure 1G,H). Drought significantly decreased the shoot biomass, root biomass and total biomass of *L. davurica* but increased the root:shoot ratio (Figure 1C–F).

### 3.2. Plant Hormones 

**Figure 2:**
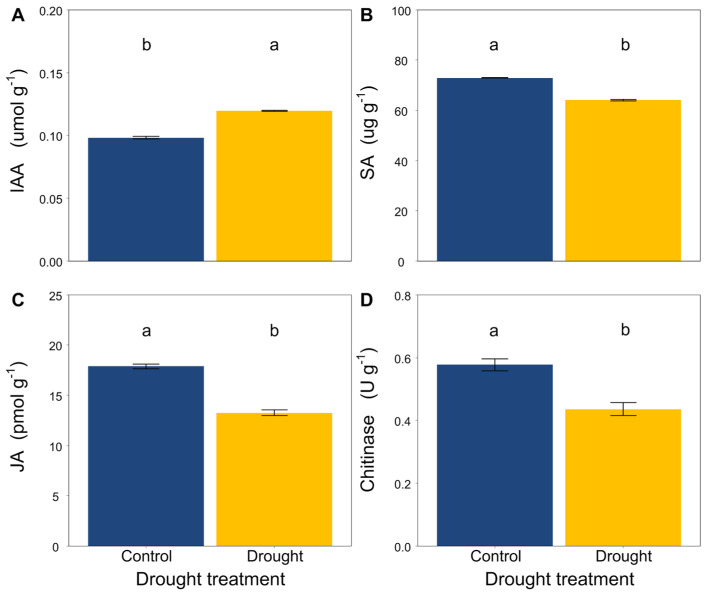
Effects of drought on (**A**) IAA, (**B**) SA, (**C**) JA content and (**D**) chitinase activity in roots of *L. davurica*. Mean ± SE are shown (n = 12). Within each panel, different lowercase letters indicate significant differences at *p* < 0.05 (Tukey’s HSD).

Drought significantly increased the contents of IAA in the *L. davurica* roots (Figure 2A), while it decreased the contents of SA and JA and the activity of chitinase (Figure 2B–D).

### 3.3. Soil Nutrients and Microbial Biomass

**Figure 3:**
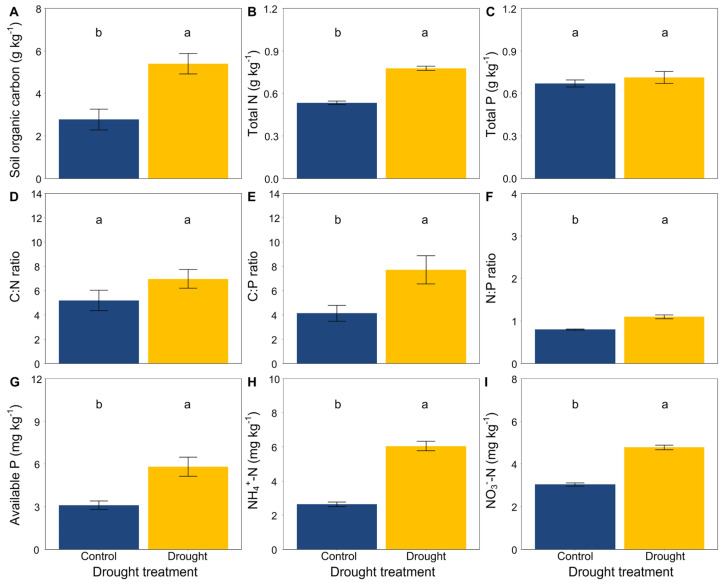
Effects of drought on (**A**) soil organic carbon, (**B**) total N, (**C**) total P, (**D**) the C:N ratio, (**E**) the C:P ratio, (**F**) the N:P ratio, (**G**) available P, and (**H**,**I**) inorganic N (NH_4_^+^-N and NO_3_^−^-N). Mean ± SE are shown (n = 12). Within each panel, different lowercase letters indicate significant differences at *p* < 0.05 (Tukey’s HSD).

**Figure 4:**
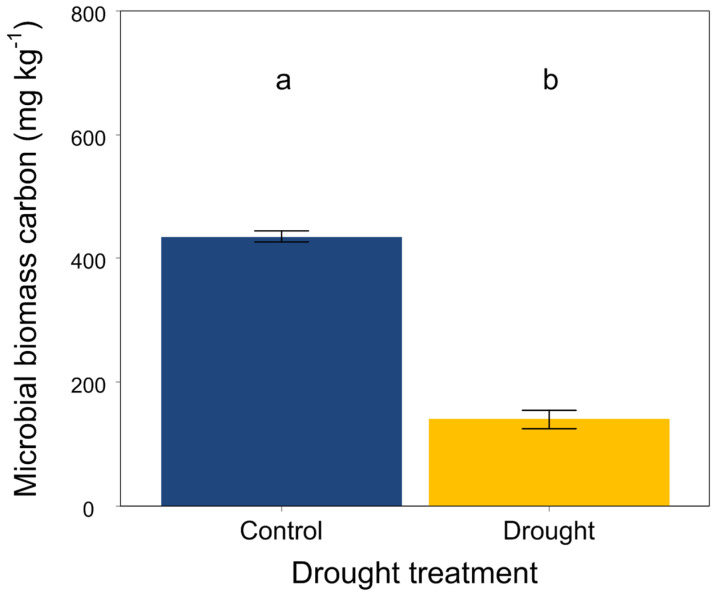
Effects of drought on soil microbial biomass carbon. Mean ± SE are shown (n = 12). Different lowercase letters indicate significant differences at *p* < 0.05 (Tukey’s HSD).

At the end of the experiment, drought significantly increased the soil organic carbon, total N, available P, NH_4_^+^-N and NO_3_^−^-N (Figure 3A,B,G–I) but did not affect the soil total P (Figure 3C). Moreover, drought had an obvious effect on the soil stoichiometric characteristics. The ratios of C:N, C:P and N:P were significantly higher in drought conditions than that in the control (Figure 3D–F). In addition, drought significantly decreased the soil microbial biomass carbon (Figure 4).

### 3.4. The Chao1 and Shannon Diversity Indices of Soil Microbial Communities

**Figure 5:**
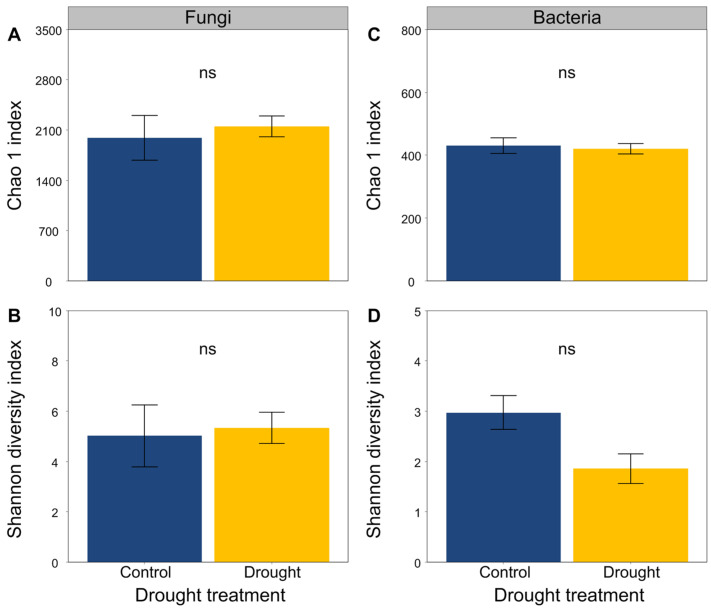
Effects of drought on (**A**,**B**) fungal and (**C**,**D**) bacterial community diversity. Mean ± SE are shown (n = 3). ns indicates no significant differences.

The soil fungal and bacterial sequences clustered into a total of 772 and 2961 OTUs, respectively. Drought did not obviously alter the Chao1 and Shannon diversity indices of the soil fungal and bacterial communities (Figure 5).

### 3.5. The Composition of Soil Microbial Communities

**Figure 6:**
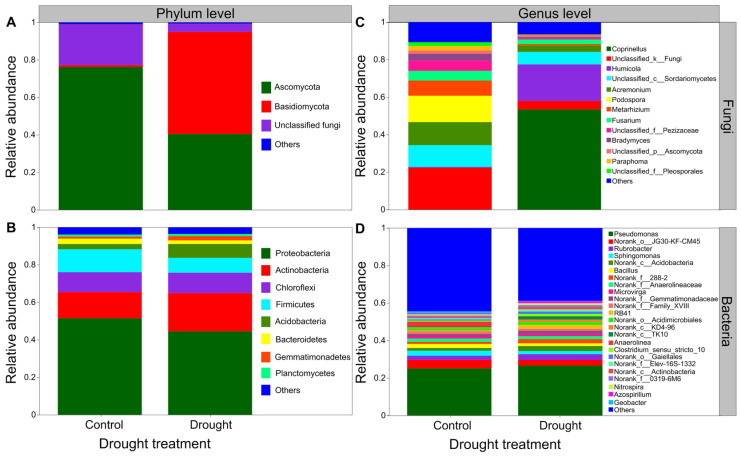
Effects of drought on the composition of fungal and bacterial community. The relative abundance of (**A**) soil fungal and (**B**) bacterial community at the phylum level, (**C**) soil fungal and (**D**) bacterial community at the genus level (n = 3).

**Figure 7:**
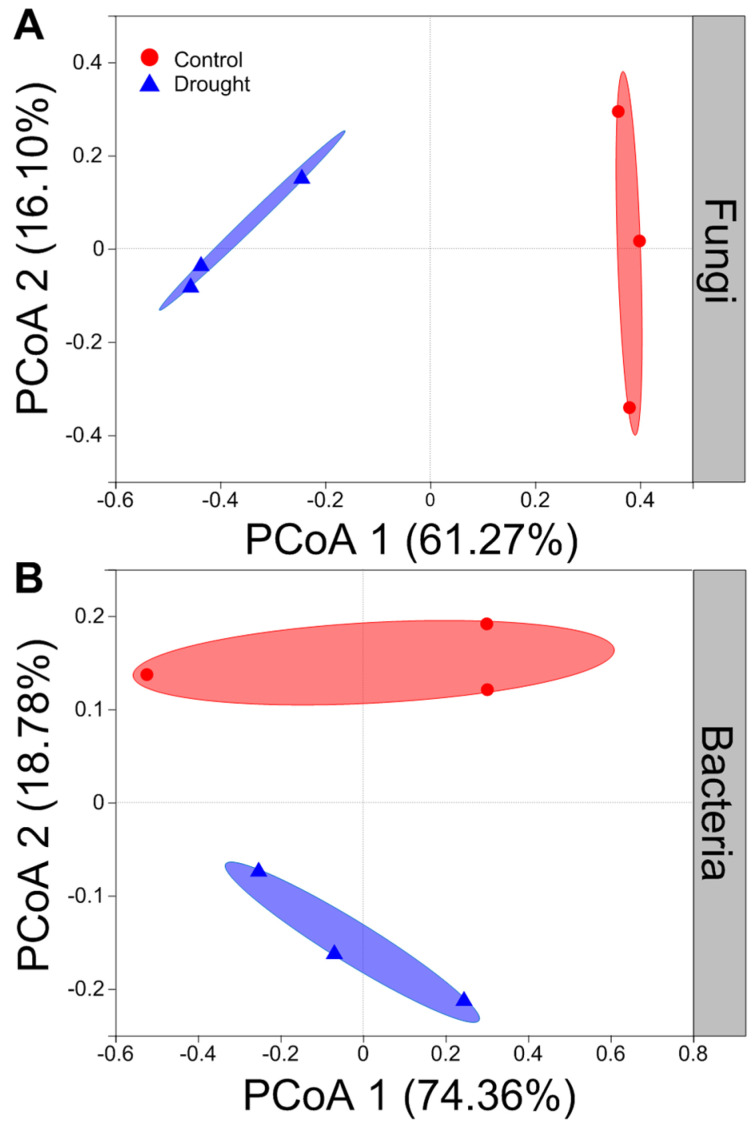
Effects of drought on the structure of fungal and bacterial community. Principal coordinates analysis (PCoA) of (**A**) fungal and (**B**) bacterial community at the operational taxonomic units (OTUs) level based on the Bray–Curtis dissimilarities (n = 3).

Drought strongly altered the composition of soil fungal communities at both the phylum and/or genus levels (Figure 6A,C). Drought clearly decreased the relative abundance of Ascomycota but increased that of the Basidiomycota (Figure 6A). Interestingly, drought strongly increased the relative abundances of *Coprinellus* and *Humicola* but decreased those of *Podospora* and *Acremonium* (Figure 6C). In contrast, drought did not clearly alter the composition of soil bacterial communities at both the phylum and/or genus levels (Figure 6B,D). Proteobacteria was the most abundant phylum between the drought and control conditions (Figure 6B). Drought slightly decreased the relative abundances of Proteobacteria and Firmicutes but increased those of Actinobacteria and Acidobacteria to some extent (Figure 6B). Moreover, drought increased the relative abundances of *Acidobacteria*, *Pseudomonas* and *Rubrobacter* to some degree but mildly decreased those of *Sphingomonas* and *Bacillus* (Figure 6D). The results of rarefaction curves indicated that the number of reads was enough to detect most of the types of soil bacterial and fungal sequences from the soil samples since the curves arrived at balanced plateaus (Figure S2). In addition, the results of PCoA showed that the composition of soil fungal and bacterial communities was clearly separated between the drought and control (Figure 7).

### 3.6. Relationships between the Soil Microbial Communities and Soil Properties

**Figure 8:**
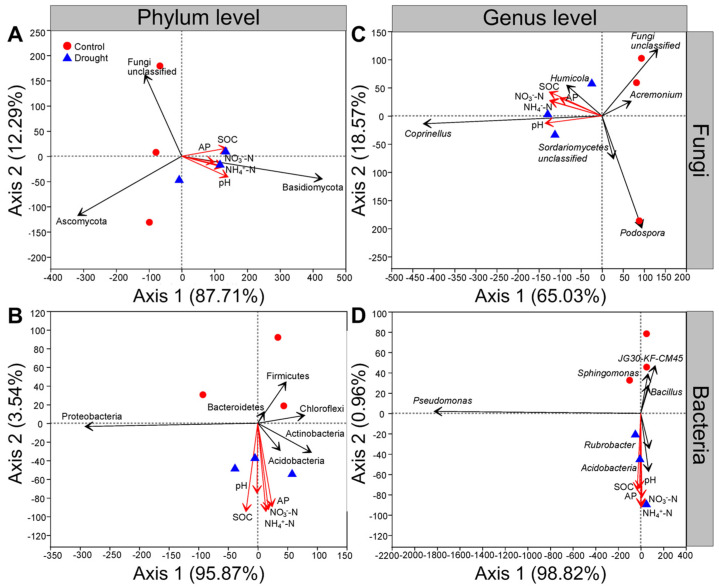
Effects of drought on the relationships among soil microbial communities and soil properties. Redundancy analysis (RDA) of relative abundance of (**A**) soil fungal or (**B**) bacterial community and soil properties at the phylum level, (**C**) soil fungal or (**D**) bacterial community and soil properties at the genus level after *L. davurica* grown in the glasshouse under drought treatment. The black solid line indicates fungal and bacterial phyla or genera, and the red solid line indicates soil properties. Soil properties indicated include soil organic carbon (SOC), available phosphorus (AP), ammonium nitrogen (NH_4_^+^-N), nitrate nitrogen (NO_3_^−^-N) and pH.

At the phylum level, the first and second axes of RDA explained 87.71% and 12.29% of the variance for fungal communities, respectively; soil organic carbon, soil pH, available P, NH_4_^+^-N and NO_3_^−^-N were positively related to the abundance of Basidiomycota but negatively related to that of Ascomycota under drought conditions (Figure 8A). The first and second axes of the RDA explained 95.87% and 3.54% of the variance in bacterial communities, respectively, and the abundance of Proteobacteria was positively related to the soil organic carbon and soil pH but negatively related to the soil available P, NH_4_^+^-N and NO_3_^−^-N under drought conditions (Figure 8B). Moreover, the soil organic carbon, soil pH, available P, NH_4_^+^-N and NO_3_^−^-N were positively related to the abundances of Acidobacteria and Actinobacteria but negatively related to those of Firmicutes, Chloroflexi and Bacteroidetes under drought conditions (Figure 8B).

At the genus level, the first and second axes of the RDA explained 65.03% and 18.57% of the variance for fungal communities, respectively; the abundances of *Coprinellus* and *Humicola* were positively related to the soil organic carbon, soil pH, available P, NH_4_^+^-N and NO_3_^−^-N under drought conditions (Figure 8C). In contrast, the abundances of *Podospora* and *Acremonium* were negatively related to the soil organic carbon, soil pH, available P, NH_4_^+^-N and NO_3_^−^-N (Figure 8C). The first and second axes of the RDA explained 98.82% and 0.96% of the variance in bacterial communities, respectively, and the soil organic carbon, soil pH, available P, NH_4_^+^-N and NO_3_^−^-N were positively related to the abundances of *Acidobacteria* and *Rubrobacter* but were negatively related to those of *Pseudomonas*, *Sphingomonas* and *Bacillus* under drought conditions (Figure 8D).

### 3.7. The Contribution of Drought and Soil Properties to the Variation in Soil Fungal Community 

**Figure 9:**
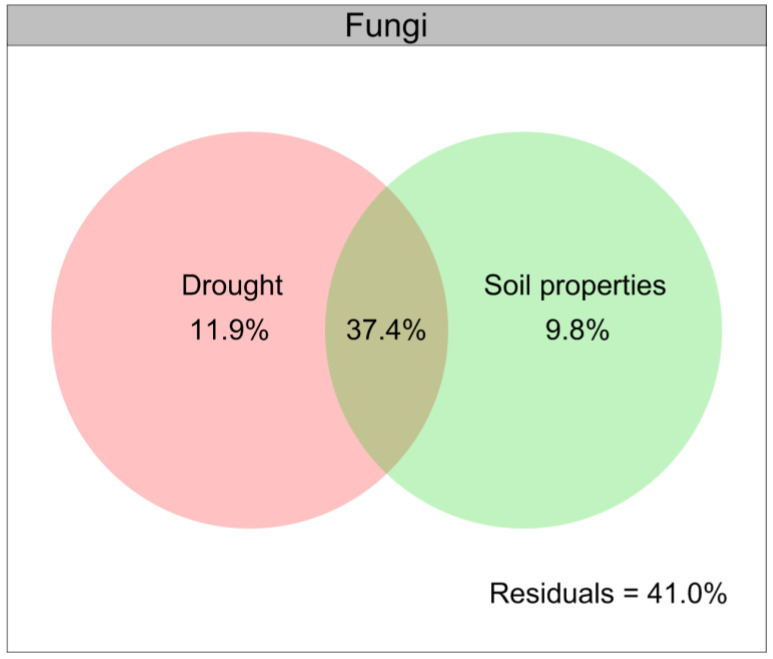
Variation partitioning analysis (VPA) of the relative contributions of drought and soil properties to variation of fungal community. Soil properties indicated include soil organic carbon, total nitrogen, total phosphorus, available phosphorus, ammonium nitrogen, nitrate nitrogen and pH.

Drought contributed a larger role of variation relative to the composition of the soil fungal community than that for the soil bacterial community (Figure 6), and the soil properties were closely related to the soil microbial communities under drought conditions (Figure 8). We further illustrated the contribution of drought and soil properties to the soil fungal community variation with a modified VPA (Figure 9). The results of VPA indicated that the complete set of all variables together explained 59.1% of the variation in the soil fungal community, and drought (11.9%) clearly contributed a larger proportion of variation of the soil fungal community compared with soil properties (9.8%) (Figure 9).

## 4. Discussion

Water-limited ecosystems are likely to be highly responsive to drought [78]. Water is considered to be one of the key environmental factors that limits the growth of plants in the Loess Plateau of northwest China [8]. Soil microbes play an important role in plant drought stress. *Lespedeza davurica* is one of the predominant species in natural semiarid grasslands in northwest China [52]. Therefore, we examined how drought alters the abiotic properties and microbial communities of soil and mediates the growth of *L. davurica*. 

In this study, drought indeed strongly altered the growth and development of *L. davurica,* including its biomass and the morphological characteristics of shoot and root. We found that drought significantly reduced the RGR, shoot biomass, root biomass and total biomass of *L. davurica*, which is consistent with previous findings that drought was the primary limiting factor for the production of biomass by *L. davurica* [53]. Under drought conditions, roots act as sensors and are responsible for resource uptake and storage, and they grow faster than leaves [79,80]. Consistent with previous research, we found that drought decreased the plant height but increased the root:shoot ratio and length of primary roots of *L. davurica*, supporting our first hypothesis that drought would increase the root biomass allocation of *L. davurica*. Root morphological traits are important for plants to manage in an environment that has limited water and/or nutrients, such as those in arid and semiarid ecosystems [53,81]. Numerous studies have shown that drought increased the root mass ratio, proportion of thin roots, root length and surface area and the numbers of root hairs [81,82,83,84,85], which help the plant to acquire nutrients from severe environments. A previous study showed that the root average diameter values of *L. davurica* are significantly affected by water levels, efficiently compete for the limited water resources and increase the acquisition of nutrients under drought conditions [53]. In this study, our findings indicated that *L. davurica* may absorb more water and soil nutrients under drought conditions by shifting its biomass allocation and morphological characteristics, which is consistent with the findings of previous studies [8,53]. However, the root vertical development could be largely constrained by the pot size as well.

Since drought limits the uptake of nutrients from the soil nutrients [86], and long-term drought is expected to alter plant physiology and metabolic pathways [56], plants are likely to alter the level of their hormones to adapt to resource-limited environments. Plant hormones play significant functions in the establishment of signaling networks to regulate stress-related responses and plant development in response to drought. Therefore, we tested whether hormones and chitinase modified the growth and development of *L. davurica* under drought conditions. Numerous reports have suggested that JA and SA have an important role in the response of plants to drought. For example, a prolonged water deficit can reduce the contents of JA and SA of common sage (*Salvia officinalis*) during leaf senescence [87]. Long-term drought decreased the contents of JA and SA in banana leaves (*Musa* spp.) [88]. Drought decreased the contents of JA and SA of two contrasting genotypes of *Catalpa bungee* [89]. Long-term drought decreased the contents of JA and SA in the roots of tea (*Camellia sinensis*) [90]. A recent study found that drought also decreased the contents of JA and SA in gray-leaved Cistus (*Cistus albidus*) seedlings [91]. In the study, we found that drought decreased the content of JA in the *L. davurica* roots, which was consistent with a previous study that the concentrations of endogenous JA first increased rapidly following drought stress and then decreased to normal levels if the stress periods were prolonged [92,93,94]. Another study also reported that severe drought slightly decreased the JA content of *Arabidopsis thaliana* [95]. In addition, a previous study also reported that SA, auxin, such as IAA, and other plant hormones interacted with JA, thus regulating the adaptation of plant to its surroundings [57]. Thus, we expected that the SA content in the *L. davurica* roots could decrease under drought as well, which was indeed confirmed by our results. Recently, a study found that prolonged drought decreased the SA content in *Brassica napus* leaves [96]. In contrast, we found that drought increased the contents of endogenous IAA in *L. davurica* roots, indicating that IAA possibly enhanced the drought tolerance of *L. davurica*. Our results were also consistent with previous findings that the application of exogenous IAA could weaken the negative effects caused by drought, and thus improved the growth of barley (*Hordeum vulgare*) [97]. In addition, we found that drought decreased the activity of chitinase in *L. davurica* roots, which was consistent with previous findings that drought decreased the activities of enzymes because of its negative effects on soil properties [98]. Similar results were reported that indicated the involvement of chitinase in the drought tolerance of tomato (*Solanum lycopersicum*) when subjected to drought [58].

Drought can typically induce a reduction in the mobility of soil nutrients, leading to some substantially considerable effects on plant performance [99,100,101]. Consistent with previous findings that drought has negative effects on soil properties and increased the concentrations of soil nutrients [33,98], we found that the concentrations of soil organic carbon, total N, available P, NH_4_^+^-N and NO_3_^−^-N were higher in drought conditions compared with the control, supporting our second hypothesis that drought would reduce the mobility of soil nutrients. In contrast, a recent study reported that drought decreased the contents of soil organic carbon, total N, NH_4_^+^-N and NO_3_^−^-N in topsoil (0–10 cm) compared with ambient water in alpine grassland [102]. The difference in this study could by owing to use of a pot experiment in a greenhouse that utilized poor quality soil from a semiarid grassland, which could have resulted in the depletion of soil nutrients.

Furthermore, drought can alter the activity, abundance, and community structure and composition of soil microbes by altering the availability of soil water and nutrients [15,24,25,27,30,103,104], which, in turn, may influence plant performance [29,32,33]. Indeed, our results indicated that drought altered the structure and composition of soil fungal and bacterial communities. Soil microbes are more likely to aggregate to avoid death or dehydration under drought conditions [105]. Consistent with previous findings that drought reduced the diffusion of substrates, microbial activities and biomass [106], we found that drought significantly decreased the soil microbial biomass carbon, because the microbial population of soil would decline considerably if the soil water content was reduced to less than a particular level [14,107]. There is no consensus about the response of soil microbial diversity to drought, although many studies have focused on the responses of soil microbes to drought [103,108,109]. In this study, we found that drought altered the structure and composition of soil microbial communities, partially supporting our third hypothesis that drought would have negative effects on the soil microbial communities. Drought can reduce the mobility of soil nutrients and limit the reproduction of soil microbes by reducing the supply of substrates and the availability of soil water to them [110,111] and decreasing soil microbial diversity [112,113,114,115]. In addition, several meta-analyses indicated that drought had a negative effect on soil microbial diversity [105,116,117,118,119]. In this study, we found that drought tended to decrease the Chao1 and Shannon diversity indices of soil bacteria but increase them in soil fungi to some extent, suggesting that drought could have a negative effect on the diversity of soil fungal communities [119]. Drought increases the fungal richness but does not alter the bacterial richness, for the reason that fungi are thought to have a greater ability to cope with drought than bacteria, owing to their capability to cumulate osmoregulatory solutes to protect their filamentous structure and metabolism [113,120,121]. This was partly consistent with our results that drought strongly altered the composition of soil fungal communities but did not significantly affect the composition of soil bacterial communities. Recently, a study also found that drought was related with an increase in the Gram-positive:Gram-negative bacteria ratio [101]. Soil fungi may remain active at a very low content of soil water compared with soil bacteria [107,116]. Since fungi can produce a large mass of hyphae that improves the transfer of moisture across long distances [114], they are likely to be more tolerant to drought compared with bacteria. Moreover, the fungi:bacteria ratio has a positive relationship with soil water content, and fungi may easily have been water-restricted compared to bacteria [45,122,123]. Besides, specialized microbes show some different responses to global change factors effects compared with fungi and bacteria [118]. For example, specialized microbes may play some key soil functions (such as ammonia oxidizer, methanotrophic, diazotrophic, phosphorus mineralizer, etc.), which are vulnerable to diversity loss owing to their lower richness [124]. The root system may improve diversity of microbes, most of which (e.g., *Pseudomonas*, *Bacillus*) can synthesize phytohormones, hydrolase enzymes or siderophores, helping plants to cope with abiotic stress (such as drought). In contrast, our results indicated that drought mildly decreased the relative abundances of *Bacillus*. Plant resistance was improved by the root-associated bacterial microbiome to drought by water stress-induced promotion capability as well [125]. Mycorrhizal fungi also can help plants for water and nutrients uptake through extraradical hyphae [126]. However, in this study, we did not find changes in these specialized microbes, possibly because the soil samples used for high-throughput sequencing were from non-rhizosphere. Furthermore, fungi tend to have slow turnover rates and utilize nitrogen-poor substrates, while bacteria are characterized by high nutrient requirements and usually dominate in soil habitats that contain high-quality substrates, such as those with lower C:P and C:N ratios [36,127,128]. In this study, we found that drought increased the C:P and C:N ratios in soils, which would be likely to favor fungal growth compared with that of bacteria. In addition, one study indicated that soil fungi are more sensitive to the wetting–drying cycle than soil bacteria under drier conditions [129]. However, another study concluded that there was no difference [130]. 

Although drought did not significantly affect the soil microbial diversity in this study, we found that drought clearly altered the composition of soil microbes. We found that the composition of soil fungal communities was more influenced by drought than bacteria, regardless of the phylum or genus level. A previous study suggested that Bacteroidetes and Proteobacteria are sensitive to drought, while Actinobacteria and Firmicutes are resistant to drought [131], which was not entirely consistent with our results. We found that drought decreased the relative abundances of Ascomycota, Proteobacteria and Firmicutes but increased those of Basidiomycota, Actinobacteria and Acidobacteria at the phylum level, which is consistent with previous findings that drought increased the relative abundance of Actinobacteria [132]. This is because soil microbes may show quite different responses to drought based on their adaptation to specific environmental conditions [42,133]. In addition, we found that drought strongly increased the relative abundances of *Coprinellus* and *Humicola* but decreased those of *Podospora* and *Acremonium* at the genus level. Moreover, we found that drought increased the relative abundances of *Acidobacteria*, *Pseudomonas* and *Rubrobacter* to some degree but mildly decreased those of *Sphingomonas* and *Bacillus*. Taken together, these results imply that the adaptation of key soil fungi and/or bacteria are important for plants to manage drought [132]. 

Moreover, we found that drought indirectly affected the diversity and composition of soil microbial communities by altering the soil physical and chemical factors, particularly the soil organic carbon, soil pH, available P, NH_4_^+^-N and NO_3_^−^-N, which were consistent with previous findings that the availability of water affected the soil microbial communities by altering the availability of soil pH and nutrients [118,134,135,136,137]. In particular, we found that the abundances of Basidiomycota, Proteobacteria, Acidobacteria and Actinobacteria were closely positively related to the soil organic carbon and soil pH under drought conditions, while the soil available P, NH_4_^+^-N and NO_3_^−^-N were closely positively related to those of Ascomycota, Chloroflexi, Firmicutes and Bacteroidetes. Given that the soil fungal community could potentially play an important role in mediating the growth of *L. davurica* in response to drought, the results of VPA further indicated that drought contributed a larger proportion of variation to the soil fungal β-diversity compared with the soil properties, thus indicating a stronger effect of drought in driving the soil fungal community.

## 5. Conclusions

This study first revealed the effects of drought on *L. davurica* owing to changes in the soil microbial communities and the availability of nutrients. Our findings demonstrated that drought considerably altered the performance and endogenous hormones of *L. davurica*, soil physicochemical properties, soil microbial biomass, and the composition of soil fungal and bacterial communities. The abundances of *Coprinellus* had the strongest positive relationship with the soil organic carbon, soil pH, available P, NH_4_^+^-N and NO_3_^−^-N under drought conditions, and possibly mediating the response of *L. davurica* growth to drought. Drought clearly contributes a larger proportion of variation relative to soil properties to soil fungal communities. An additional benefit of this study is its analysis of forage production and natural grassland vegetation recovery in semiarid regions of the Loess Plateau of northwest China. However, future experiments under natural conditions are necessary to fully understand how drought alters the abiotic and biotic properties of soil to regulate the growth of *L. davurica*.

## Data Availability

The fungal and bacterial raw DNA sequences used in this study have been deposited in the Sequence Read Achieve (SRA) of the NCBI database under the accession number PRJNA755514 for open access.

## Supplementary Materials

The following supporting information can be downloaded at: https://www.mdpi.com/article/10.3390/jof8040384/s1, Figure S1: Effects of drought on (A) plant height and (B) length of primary root of *Lespedeza davurica*; Figure S2. Rarefaction curves of OTUs for (A) fungal and (B) bacterial communities. Table S1: Basic concentrations of soil organic carbon (SOC), total nitrogen (TN), total phosphorus (TP), available phosphorus (AP), inorganic nitrogen (NH_4_^+^-N and NO_3_^−^-N) and pH before the sowing in glasshouse.
