# Supplementary material for: Effects of Drought on the Growth of Lespedeza davurica through the Alteration of Soil Microbial Communities and Nutrient Availability
Source: J Fungi (Basel). 2022 Apr 10;8(4):384. doi: 10.3390/jof8040384 (PMC9025084; doi:10.3390/jof8040384)
Supplement: Supplementary file 1 [file jof-08-00384-s001.zip › jof-1655200-supplementary.pdf]

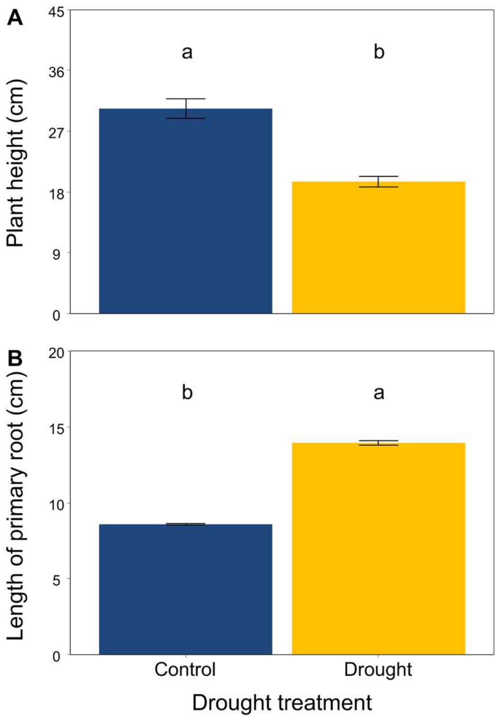

Figure S1. Effects of drought on (**A**) plant height and (**B**) length of primary root of *Lespedeza davurica*. Mean ± SE are shown (n = 12). Within each panel, different lowercase letters indicate significant differences at $p < 0.05$ (Tukey's tests).

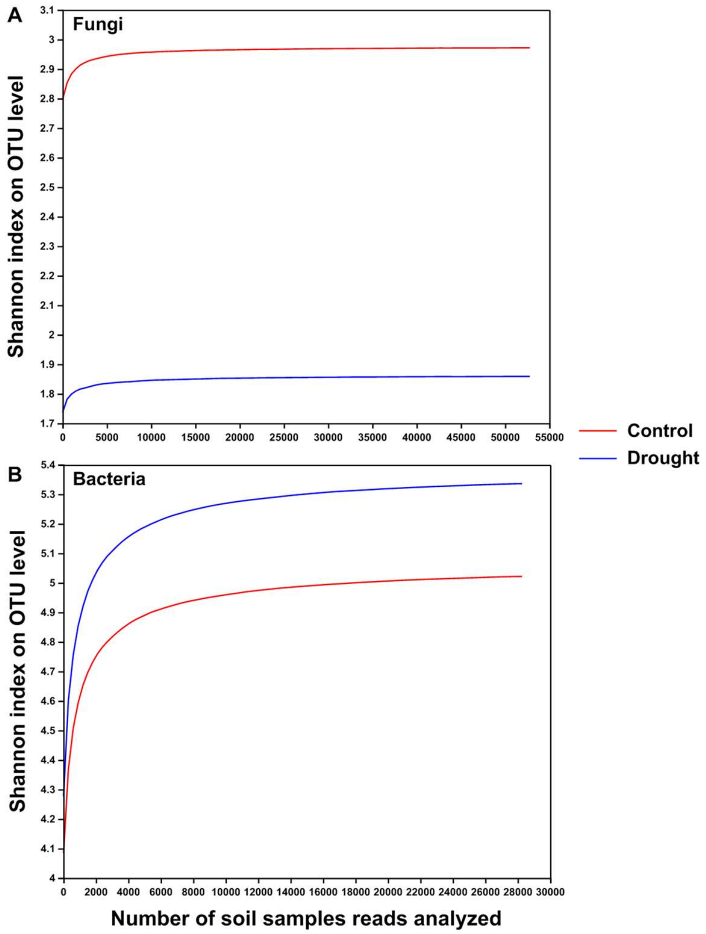

Figure S2. Rarefaction curves of OTUs for (**A**) fungal and (**B**) bacterial communities.

Table S1. Basic concentrations of soil organic carbon (SOC), total nitrogen (TN), total phosphorus (TP), available phosphorus (AP), inorganic nitrogen (NH_4^+-N and NO_3^--N) and pH before the sowing in glasshouse. Data are represented as the mean of four replicates with standard errors in brackets.

SOC (g/kg)	TN (g/kg)	TP (g/kg)	AP (mg/kg)	NH_4^+-N (mg/kg)	NO_3^--N (mg/kg)	pH
5.46 (0.42)	0.83 (0.05)	0.75 (0.03)	3.84 (0.06)	9.89 (0.88)	5.12 (0.08)	8.25 (0.03)
